# Meta-analysis of cancer risk among end stage renal disease undergoing maintenance dialysis

**DOI:** 10.1515/biol-2022-0553

**Published:** 2023-02-17

**Authors:** Xin Xie, Fang Li, Longsheng Xie, Yanxia Yu, Santao Ou, Rongfang He

**Affiliations:** Department of Nursing, The Affiliated Hospital of Southwest Medical University, Luzhou, China; Department of Nephrology, The Affiliated Hospital of Southwest Medical University, Luzhou, China; Sichuan Clinical Research Center for Birth Defects, The Affiliated Hospital of Southwest Medical University, Luzhou, China; Department of Cardiology, People’s Hospital of Jianyang City, Jianyang, China; Department of Psychiatry, The Affiliated Hospital of Southwest Medical University, No. 100 Taiping Street, Luzhou, Sichuan, 646000, China; Sichuan Clinical Research Center for Birth Defects, The Affiliated Hospital of Southwest Medical University, Luzhou, China

**Keywords:** maintenance dialysis, cancer incidence, meta-analysis

## Abstract

Currently, there is no consensus on whether maintenance dialysis increases cancer risk in patients with end-stage renal disease (ESRD). Therefore, this study was to systematically evaluate the risk of cancer among ESRD patients undergoing maintenance dialysis. Related studies on the impact of maintenance dialysis on cancer risk were retrieved from PubMed, Embase, Cochrane Library, and other databases from their respective inceptions to 19 February 2021. ESRD patients receiving maintenance dialysis were classified into cancer including non-melanoma skin cancer (NMSC) and cancer excluding NMSC. Standardized incidence ratio (SIR) with its 95% confidence interval (95% CI) was calculated to assess cancer risk. Fourteen studies were included in the meta-analysis. The risk of cancer in patients undergoing maintenance dialysis (with or without NMSC) was significantly higher than controls both in cancer including NMSC (SIR = 1.38, 95% CI: 1.27–1.49, *P* < 0.001) and cancer excluding NMSC (SIR = 1.34, 95% CI: 1.23–1.47, *P* < 0.001). Subgroup results identified the higher risk of cancer incidence in both men and women receiving maintenance dialysis. Meanwhile, elevated excess risks were observed among patients with younger age and shorter follow-up time (*P* < 0.001). Meanwhile, the combined SIR of bladder, cervix, colorectum, kidney, liver, thyroid, tongue, and other cancers were all increased (*P* < 0.05). ESRD patients undergoing dialysis has higher risk of cancer.

## 1 Introduction

For patients with end stage renal disease (ESRD), chronic hemodialysis and renal replacement therapies are conventional treatment strategies. Following cardiovascular disease and infection, cancer became one of the most common reasons for death among ESRD patients. Although the underlying mechanism for the occurrence of cancers has not yet been fully clarified, several hypotheses related with dialysis factors has been put forward, such as altered DNA repair and methylation [1], uremia-induced immune dysfunction [2], and elevated specific carcinogen levels [3].

The investigation of cancer risk among patients undergoing dialysis has been widely designed; however, the conclusion remains inconsistent. A previous study involving 52,105 dialysis patients confirmed the higher cancer incidence rate as compared with non-dialysis patients, and the risk of cancers was dominated in younger and female patients [4]. Among Asian populations, 2,352 newly diagnosed cancers out of 40,833 ESRD patients who underwent maintenance dialysis have been reported [5], and elevated cancer occurrence was among older, male, and chronic liver disease. Furthermore, previous evidence showed controversial conclusion on the risk of specific cancer. For example, Butler et al. demonstrated that significantly elevated risk of cancers of the kidney/renal pelvis and bladder [6]. However, studies have reported that maintenance dialysis would significantly increase the risks of kidney cancer, skin non-melanoma cancer, and oral cavity cancer [7,8].

Of the various studies researching on the analyzed cancer in ESRD patients, skin cancer was rarely investigated. Moreover, in many countries, non-melanoma skin cancer (NMSC) has not been included in cancer registries [9,10]. Thus, in our study, the maintenance dialysis patients were classified into cancer including NMSC and cancer excluding NMSC. Furthermore, subgroup analysis stratified by gender, first dialysis age, follow-up time, and region was designed to systematically evaluate the risk of cancer among patients undergoing maintenance dialysis. Additionally, according to the type of cancer, the standardized incidence ratio (SIR) with its 95% confidence interval (95% CI) corresponding to each cancer was also summarized. Finally, our data showed ESRD patients undergoing dialysis had higher risk of cancer, especially among younger patients and those undergoing shorter time of dialysis.

## 2 Methods

### 2.1 Selection strategy

A literature search was performed in PubMed, Cochrane Library, and Embase databases without language restriction. The following key words were used in the study selection: dialysis, hemodialysis, renal dialysis, neoplasms, cancer, carcinoma, “cohort study” OR “cross sectional studies” OR “case control studies” till 19 February 2021. The search strategy in the three databases is shown in Tables A1–A3. Moreover, in order to enroll more research studies, print-out literatures were also searched. Additionally, we further retrieved the references of included articles and reviews.

### 2.2 Study selection

The studies including prospective, retrospective cohort studies, case–control studies, cross-sectional studies, and other research types without language limitations in accordance with the following inclusion criteria would be included: [1] the subjects of the study are adult (≥18 years old) patients undergoing maintenance dialysis lasting for 3 months or more, [2] the outcomes of patients are cancers, and [3] the literature reports one or more of the following outcomes: SIR (the ratio of observed to expected cancers) of all cancers, SIR of each subgroup (such as gender, age of first dialysis, follow-up time, region, etc.), and the specific SIR of each type of cancer.

Exclusion criteria included: [1] patients who have had cancer or kidney transplantation before dialysis treatment, [2] the non-research articles, such as reviews, comments, and conference summaries, [3] the studies that lack sufficient data for meta-analysis due to incomplete data, and [4] duplicated studies or same data used in multiple articles (only included the study with the most complete information).

### 2.3 Data extraction

Based on the designed criteria, studies were screened by two investigators (X.X. and F.L.) independently. According to the standardized form, the following information were extracted: the basic information of the included study, including the first author, study area, year of publication, basic characteristics of the study object (including sample size, age, gender, etc.), follow-up time, and outcome indicators.

After both of them have completed the above data extraction work, they would exchange the review and extraction forms. If there were any inconsistencies, they would discuss with the third person (R.F.H.).

### 2.4 Statistical analysis

The cancers were divided into two categories: cancer including NMSC and cancer excluding NMSC. SIR with its 95% CI extracted from the original text were combined to investigate the risk of cancers. Heterogeneity among individual studies was assessed using Cochran’s *Q* test and *I* ^2^ test [11]. *P* < 0.05 and/or *I* ^2^ > 50% suggested obvious heterogeneity existed among the studies, and the random effects model would be selected to calculate the pooled data; otherwise, the fixed effect model was selected (*P* ≥ 0.05 and *I* ^2^ ≤ 50%).

Subgroup analysis stratified by gender, first dialysis age, follow-up time, and region was designed both in the group of cancer including NMSC and cancer excluding NMSC to explore the influence of these factors on heterogeneity and combined results. In addition, the SIR (95% CI) corresponding to each cancer was summarized according to the type of cancer.

Sensitivity analysis was performed through omitting one study each time to investigate the stability of the results. Finally, the Egger test was used to evaluate whether there was significant publication bias among the included studies. All statistical analysis was performed using Stata12.0 software.

## 3 Results

### 3.1 Studies selection

**Figure 1:**
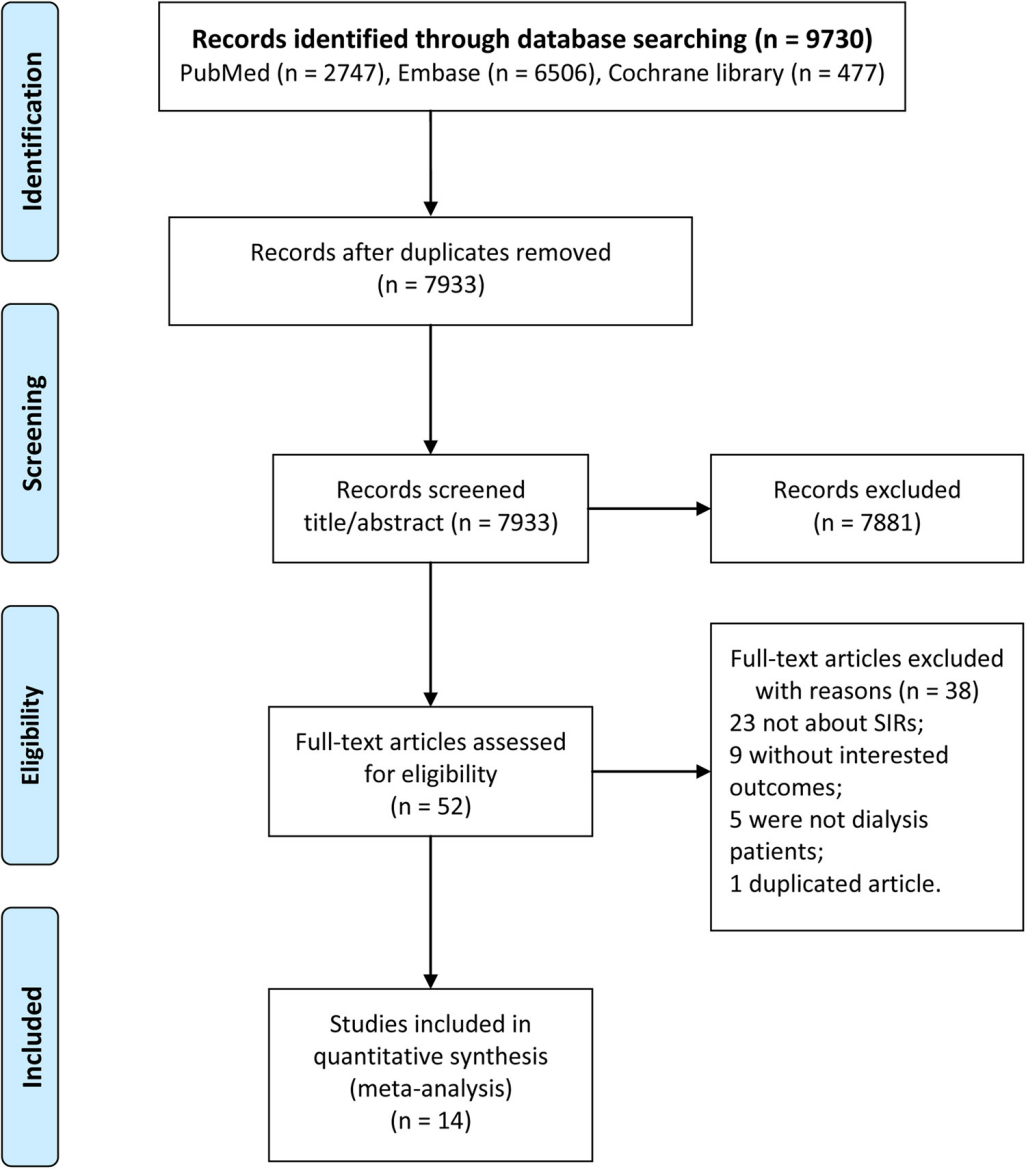
The detailed flow chart of study selection.

The detailed information associated with search process is shown in Figure 1. In this study, a total of 9,730 studies were first searched, including 2,747 articles in PubMed, 6,506 articles in Embase, and 477 articles in the Cochrane library. After removing 1,797 duplicated documents, there were 7,933 articles remaining. We excluded 7,881 articles after browsing the titles and reading the abstract. Then, total 52 articles were fully reviewed and 38 articles were excluded (23 studies did not provide SIR, nine studies did not provide interested outcomes, five studies did not involve dialysis patients, and one study was duplicated article). No study was screened out from print-out literatures. Finally, 14 articles were included in this meta-analysis [6,12–24].

### 3.2 Characteristics of included studies

As shown in Table 1, a total of 14 cohort studies were included in this meta-analysis [6,12–24]. The included studies were published from 1989 to 2017 and researched in various countries, including China, America, Japan, as well as Italy. In total, 1,596,525 subjects including 867,964 males and 728,561 females with median age 48.7–70 were included in the study, and the size of subjects in each study ranged from 912 to 521,404. These subjects were followed up 2.3–4.9 years. Among them, the study by Lee et al. [17] did not report the SIR of cancer, but reported the risk of cancer at different follow-up times. The study by Wang et al. [23] only reported the SIR of skin cancer (NMSC, melanoma). The data of three cohorts (Australia and New Zealand, Europe, United States) were reported in the study by Maisonneuve et al. [20], and three studies also reported SIR (95% CI) for both cancers including NMSC and cancer excluding NMSC [12,14,16].

**Table 1:** Characteristics of 14 included studies in this meta-analysis

Study	Country	Study period	*n*, M/F	Age, years	Mean follow-up time, years	Person- years	Cancer including NMSC	No. of cancer	SIR (95% CI)
Birkeland et al., 2000	Denmark	NR-1995	3,592, 2,154/1,438	50.2 ± 17.2	2.3 ± 2.5	8,043	Yes	110	1.40 (1.15, 1.68)
							No	77	1.16 (0.92, 1.45)
Butler et al., 2015	United States	1996–2009	482,510, 259,206/223,304	Median 67	Median 2.5	988,395	No	35,767	1.42 (1.41, 1.43)
Cheung et al., 2016	China	1994–2014	6,254, 3,403/2,851	64.0 ± 13.0	2.4 (0.16, 20.1)	14,887	Yes	220	1.44 (1.26, 1.65)
Hortlund, M 2017	Denmark	1977–2011	24,698, 15,346/9,352	Mean 61	3.2	79,881	Yes	1,713	1.55 (1.48, 1.62)
							No	1,463	1.40 (1.33, 1.47)
Lee et al., 2009	Korea	1996–2005	4,562, 2,760/1,802	50.7 ± 14.6	3.6 ± 3.9	NR	No	106	NR
Lin et al., 2012	China	1997–2008	92,348, 44,825/47,523	60.4 ± 14.8	4.4	409,909	Yes	4,328	1.4 (1.3, 1.4)
Loy et al., 2013	Singapore	1998–2007	5,505, 2,875/2,630	58.1 ± 13.0	Median 3.9	NR	No	267	1.66 (1.42, 1.95)
Maisonneuve et al., 1999	Australia and New Zealand	1980–1994	13,497, 7,513/5,984	Mean 48.7	2.5	34,456	No	500	1.8 (1.7, 2.0)
	Europe		296,903,173,375/123,528	Mean 51.8	2.2	858,532	No	6,849	1.10 (1.07, 1.13)
	United States		521,404, 278,348/243,056	Mean 57.8	2.9	1,152,047	No	17,695	1.2 (1.18, 1.22)
Mazzucotelli et al., 2017	Italy	1997–2012	912, 597/315	Median 55.5	2.5 (1.4, 3.5)^$^	2,400	Yes	24	1.4 (0.9, 2.1)
Port et al., 1989	United States	1973–1984	4,161, 2,363/1,798	Mean 52	NR	NR	No	63	1.12 (0.86, 1.43)
Stewart et al., 2009	Australia	1982–2003	23,764, 12,833/10,931	Mean 54.5	2.7	63,431	No	1,018	1.45 (1.36, 1.54)
Taborelli et al., 2019	Italy	1998–2013	3,407, 2,126/1,281	70 (6,177)^$^	2.3 (1.0, 4.5)^$^	10,798	Yes	330	1.29 (1.15, 1.43)
							No	249	1.17 (1.03, 1.32)
Wang et al., 2016	China	2000–2013	79,668, 39,698/39,970	64.0 ± 13.2	4.95 ± 3.48	394,109	NMSC	248	1.58 (1.39, 1.79)
							Melanoma	22	1.44 (0.91, 2.19)
Yoo et al., 2017	Korea	2008–2014	5,235, 3,070/2,165	56.3 ± 13.2	NR	7,835.07	Yes	66	0.94 (0.73, 1.20)

F, female; M, male; NMSC, non-melanoma skin cancer; NR, not reported; SIR, standardized incidence ratio; ^$^median (interquartile range).

### 3.3 Higher overall cancer risk in patients receiving maintenance dialysis

**Figure 2:**
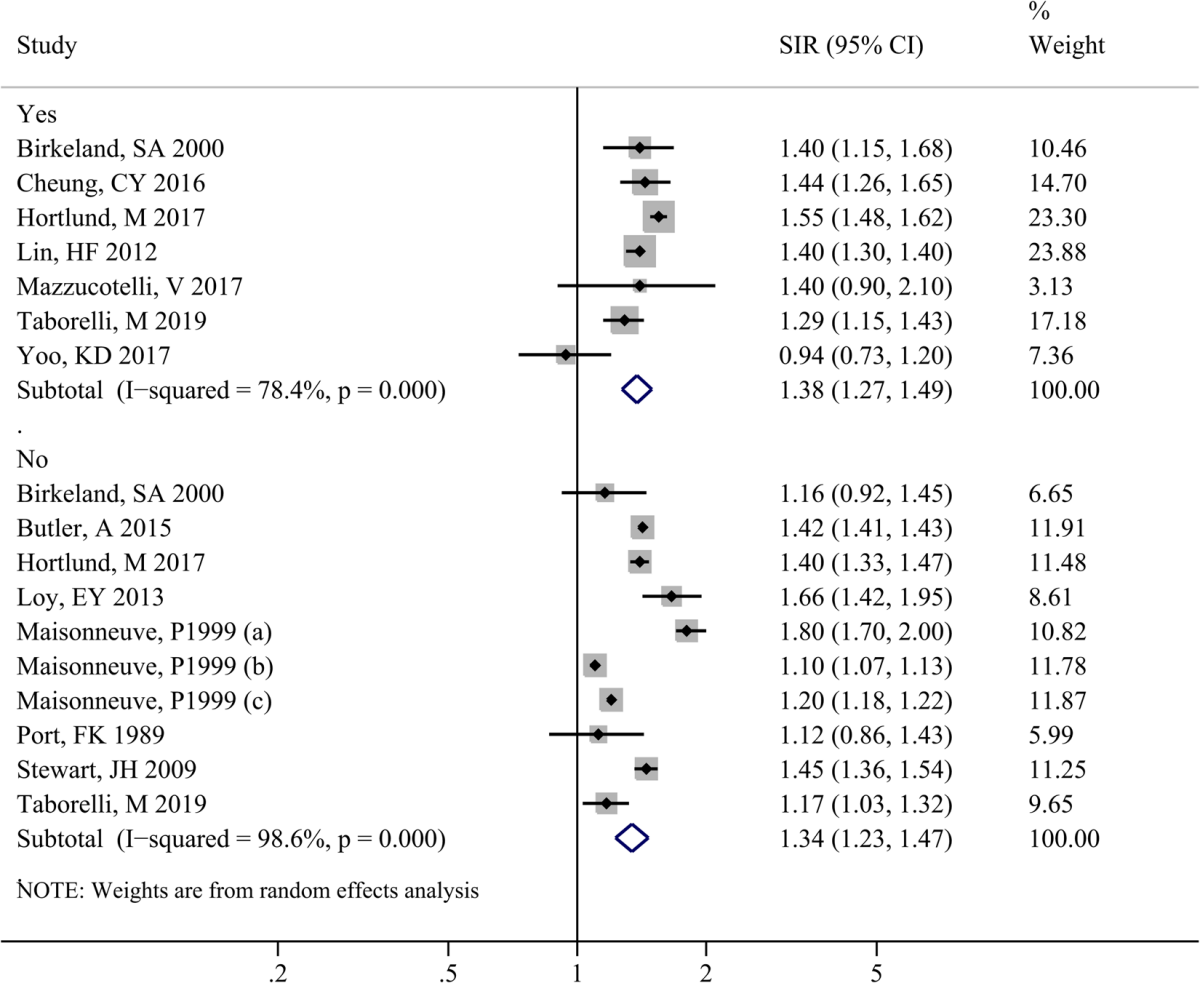
Forest plot for meta-analyzing the risk of cancer among maintenance hemodialysis patients.

As shown in Figure 2, seven studies reported the risk of cancer including NMSC, and ten studies reported the risk of cancer excluding NMSC. Significant heterogeneity was observed both in the two groups (cancer including NMSC: *I* ^2^ = 78.4%, *P* < 0.001; cancer excluding NMSC: *I* ^2^ = 98.6%, *P* < 0.001), thus the random effects model was chosen for meta-analysis. Results showed that the risk of cancer in maintenance dialysis patients (regardless of whether NMSC was included) was significantly higher than the predicted value both in cancer including NMSC (SIR = 1.38, 95% CI: 1.27–1.49, *P* < 0.001) and cancer excluding NMSC (SIR = 1.34, 95% CI: 1.23–1.47, *P* < 0.001).

### 3.4 Cancer risk assessment in subgroup analysis

As shown in Table 2, significant higher risk of cancer in both men (SIR = 1.32, 95% CI: 1.17–1.48, *P* < 0.001) and women (SIR = 1.57, 95% CI: 1.47–1.67, *P* < 0.001) in the group of cancer including NMSC, and the difference between the two groups was significant (*P* = 0.010). Although significant higher risk of cancer in both males (SIR = 1.26, 95% CI: 1.08–1.49, *P* = 0.05) and females (SIR = 1.50, 95% CI: 1.28–1.76; *P* < 0.001), the difference on the risk of cancer incidence between the two groups was not significant (*P* = 0.140).

**Table 2:** Subgroup analyses of overall cancer risk in dialysis patients

Subgroup	Cancer including NMSC					Cancer excluding NMSC				
	No. studies	Pooled SIR (95% CI)	*P*-value^A^	Heterogeneity		No. studies	Pooled SIR (95% CI)	*P*-value^A^	Heterogeneity	
				*I* ^2^ (%)	*P*-value^H^				*I* ^2^ (%)	*P*-value^H^
Gender			0.010					0.140^#^		
Male	5	1.32 (1.17, 1.48)	<0.001	81.4	<0.001	5	1.26 (1.08, 1.49)	0.005	95.8	<0.001
Female	5	1.57 (1.47, 1.67)	<0.001	25.4	0.252	5	1.50 (1.28, 1.76)	<0.001	95.0	<0.001
Age at first dialysis, years			<0.001^#^					<0.001^#^		
18–34	1	9.20 (5.30, 16.00)	<0.001	NA	NA	4	4.09 (2.59, 6.47)	<0.001	93.0	<0.001
35–64	2	2.71 (1.58, 4.65)	<0.001	98.2	<0.001	4	1.88 (1.41, 2.50)	<0.001	96.9	<0.001
≥65	2	0.80 (0.75, 0.85)	<0.001	0.0	0.910	4	1.21 (1.11, 1.32)	<0.001	78.2	<0.001
Follow-up time, years			<0.001^#^					0.005^#^		
<1	3	8.31 (7.71, 8.96)	<0.001	0.0	0.954	5	1.95 (1.45, 2.62)	<0.001	98.6	<0.001
1–2	3	2.64 (1.64, 4.29)	<0.001	92.7	<0.001	5	1.36 (1.17, 1.58)	<0.001	94.3	<0.001
2–5	3	1.52 (1.12, 2.06)	0.001	97.6	<0.001	5	1.29 (1.09, 1.52)	0.002	95.8	<0.001
>5	3	0.76 (0.45, 1.29)	0.309	96.7	<0.001	5	1.11 (0.97, 1.26)	0.129	92.4	<0.001
Area			0.360^#^				0.014^#^			
Asian	3	1.29 (1.10, 1.53)	0.002	79.8	0.007	1	1.66 (1.42, 1.95)	<0.001	NA	NA
Non-Asian	4	1.42 (1.26, 1.61)	<0.001	70.0	0.019	9	1.32 (1.20, 1.44)	<0.001	98.8	<0.001

^A^ *P*-value for test of the association; ^H^ *P*-value for the heterogeneity within each subgroup; NA, not available; NMSC, non-melanoma skin cancer; SIR, standardized incidence ratio.

^#^ *P*-value for test of difference of subgroups.

According to the age of first dialysis, the study subjects were divided into three groups (18–34, 35–64, ≥65 year), and the difference of cancer risk between cancer including NMSC and cancer excluding NMSC were statistically significant (*P* < 0.001). The risk of cancer showed age-dependent trend (*P* < 0.001). Similarly, the combined results of follow-up time showed a similar trend (*P* < 0.001). Notably, after a follow-up time of more than 5 years, the pooling SIR (95% CI) of cancer including NMSC and cancer excluding NMSC was not statistically significant (*P* > 0.05), suggesting that the follow-up time might be one of sources for the significant heterogeneity among cancer including NMSC and cancer excluding NMSC.

Significant higher cancer risk was observed both in Asian subgroup and non-Asian subgroup (*P* < 0.001). However, the difference between the two groups was not statistically significant in cancer including NMSC (*P* = 0.360), and significant difference was calculated for cancer excluding NMSC (*P* = 0.014).

The specific cancers risk assessment is shown in Table 3, and the results showed that the risk of breast cancer, Hodgkin’s lymphoma, non-Hodgkin’s lymphoma, leukemia, lung cancer, pancreatic cancer, prostate cancer, gastric cancer, and uterine cancer were not statistically significant (*P* > 0.05). Notably, significant higher risk of bladder, cervix, colorectal, kidney, liver, thyroid, tongue and other cancers, melanoma, myeloma, and NMSC was observed as compared with healthy control (*P* < 0.05). Except for liver cancer and melanoma (*I* ^2^ > 50.0%, *P* < 0.05), there was no significant heterogeneity across the included studies (*I* ^2^ < 50.0%, *P* > 0.05).

**Table 3:** Pooled risks of specific cancer types in dialysis patients

Type/site of cancer	No. studies	Pooled SIR (95% CI)	*P*-value^A^	Heterogeneity	
				*I* ^2^ (%)	*P*-value^H^
Bladder	14	2.30 (1.78, 2.96)	<0.001	96.6	<0.001
Breast (F)	12	1.18 (0.96, 1.44)	0.124	96.8	<0.001
Cervix of uterus (F)	8	1.86 (1.26, 2.75)	0.002	91.4	<0.001
Colorectal	6	1.18 (1.02, 1.36)	0.028	80.9	<0.001
HL	5	1.53 (0.98, 2.39)	0.065	60.7	0.037
Kidney	14	4.84 (4.14, 5.65)	<0.001	92.5	<0.001
Leukemia	7	1.04 (0.64, 1.71)	0.865	94.1	<0.001
Liver	8	1.41 (1.29, 1.54)	<0.001	11.9	0.337
Lung	11	1.06 (0.87, 1.29)	0.552	97.9	<0.001
Melanoma	5	1.51 (1.22, 1.87)	<0.001	0.0	0.543
Myeloma	8	3.38 (2.54, 4.50)	<0.001	86.9	<0.001
NMSC	6	2.45 (1.36, 4.43)	0.003	97.7	<0.001
NHL	12	1.23 (0.99, 1.54)	0.066	85.3	<0.001
Pancreas	4	1.27 (0.88, 1.84)	0.209	74.8	0.008
Prostate (M)	12	0.91 (0.76, 1.09)	0.294	92.6	<0.001
Stomach	9	1.10 (0.82, 1.50)	0.522	94.7	<0.001
Thyroid	10	3.16 (2.16, 4.63)	<0.001	86.9	<0.001
Tongue	6	1.86 (1.46, 2.37)	<0.001	49.8	0.076
Uterus (F)	8	1.05 (0.82, 1.35)	0.691	59.8	0.015

^A^ *P* value for test of the association; ^H^ *P*-value for the heterogeneity within each subgroup.

F, female; M, male; HL, Hodgkin lymphoma; NHL, non-Hodgkin lymphoma; SIR, standardized incidence ratio.

### 3.5 Sensitivity analysis

**Figure 3:**
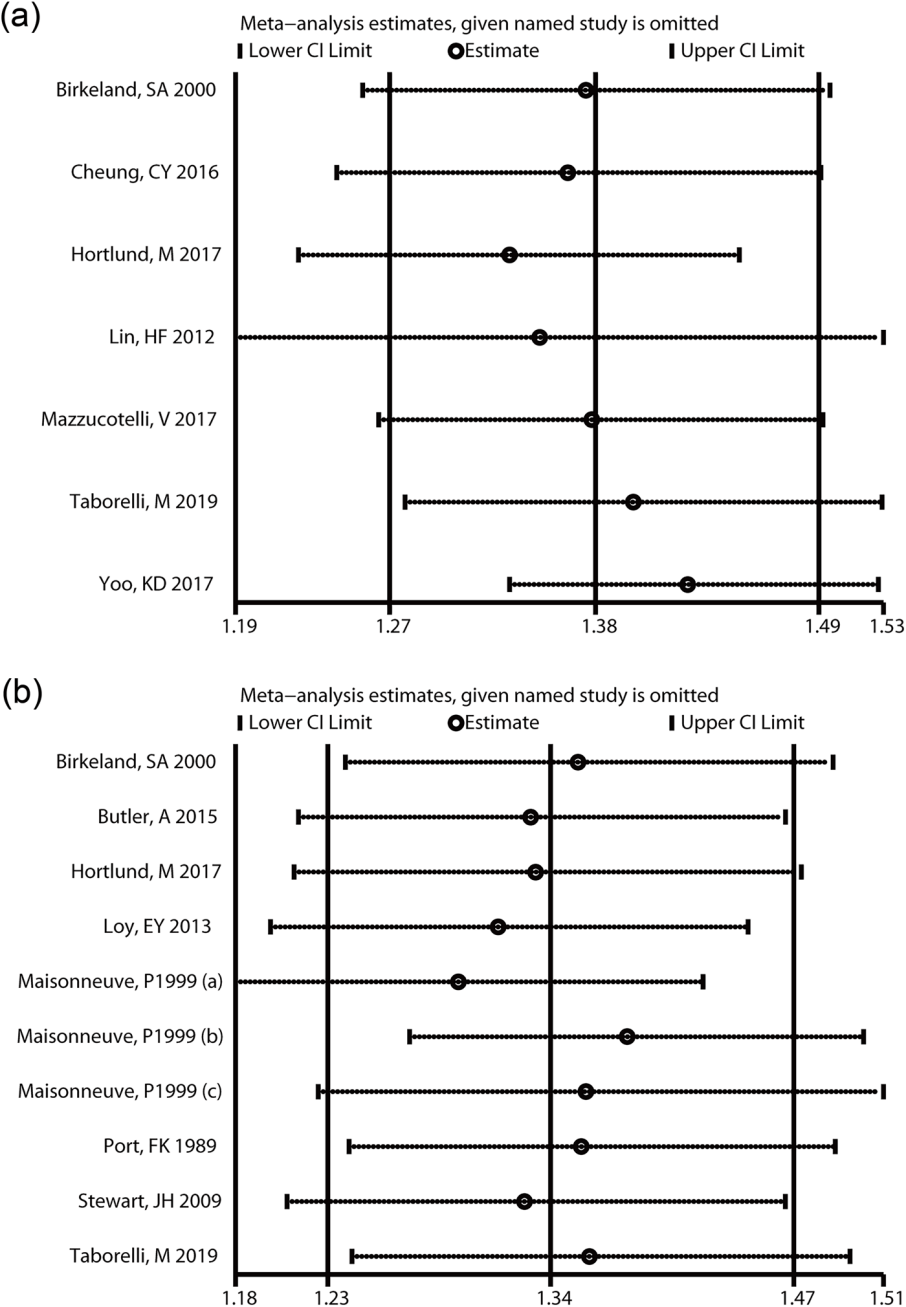
Sensitivity analysis of meta-analyzing the cancer risk among patients with cancer including NMSC and patients with cancer excluding NMSC. (a) Cancer including NMSC and (b) cancer excluding NMSC.

Sensitivity analysis of overall risk of cancer assessment was carried out. When we omitted each study among the studies researching on cancer including NMSC, the pooled SIR ranged from 1.33 to 1.43 (Figure 3a). When we omitted one study out of studies researching on cancer excluding NMSC, the pooled SIR ranged from 1.30 to 1.38 (Figure 3b), suggesting that the combined results of cancer including NMSC and cancer excluding NMSC are stable.

## 4 Discussion

In the present meta-analysis, 14 cohort studies were included. The risk of cancer in patients undergoing maintenance dialysis was significantly higher than the predicted value. Moreover, the subgroup results indicated that the risk of cancer in both men and women receiving maintenance dialysis was higher than general population. Among cancer including NMSC patients, the risk of cancer was significantly higher in female than that in male. Notably, elevated cancer risks were observed among patients with younger age and shorter follow-up time, and significantly increased risk of bladder, cervix, colorectum, kidney, liver, thyroid, and tongue were calculated among patients undergoing dialysis.

The increased cancer risks among patients undergoing dialysis had been reported previously in other industrialized countries [18–20], such as Asia [17,18], Australia/New Zealand [22], United States [6], and northern Europe [16]. Although previous evidence confirmed the higher risk of cancer development in patients undergoing dialysis, the mechanism involved in the association has not been completely clarified. The following hypothesis might be reasons for the increased cancer incidence. Uremic toxins was involved in chronic inflammation, oxidative stress damage, and immune damage, and loss of renal excretion function might lead to accumulation of carcinogenic compounds [25,26]. In addition, pro-oxidative damage in uremia might be related with the increased cancer development introduced by the activation of chronic inflammation-related complement, cytokine production, and neutrophil aggregation [27]. Furthermore, maintenance dialysis might lead to damaged DNA repair mechanism [28] and immune dysfunction [29,30]. Therefore, it is necessary for chronic dialysis patients to assess the tumor occurrence regularly in the daily clinical management.

Further, subgroup analysis demonstrated that the occurrence of cancer risk decreased significantly with the increase of dialysis age and follow-up time. The following reasons might be responsible for the age-related association. As compared with older patients, younger patients had significantly higher chance for developing cancer, and their immune defenses were more easily affected by virus-related malignancies [3,18]. Meanwhile, for elderly dialysis patients, some chronic illnesses, such as comorbidities and frailty would cause their death before cancer development. Our data showed that, until the follow-up time of more than 5 years, no significant difference on cancer risk between ESRD patients and general population was observed. Meanwhile, with the duration of dialysis, the cancer risk decreased significantly. It may be that the cancer has not caused clinical signs prior to dialysis treatment. Therefore, these data reminded us that the cancer detection should be paid more attention among younger patients and patients in the early years of dialysis.

Obvious heterogeneity was calculated in the present meta-analysis. Although subgroup analysis was designed to explore the potential source of heterogeneity, other related factors associated with cancer development could not be performed. Especially, with the development of clinical technology, various types of hemodiafiltration have been used in clinic, including routine on-line hemodiafiltration, high-flux hemodialysis, and hemofiltration. Previous data showed differential clinical efficiency among patients [31,32]. However, according to limited information, subgroup analysis stratified by hemodiafiltration types could not be performed. Thus, further study should be recommended to explore the type of hemodiafiltration on the cancer risk. However, the sensitive analysis confirmed the stability of the conclusion.

Some other limitations should also be noted in the present meta-analysis. First, it is widely known that the development of cancers are related with various independent factors, such as smoking, dietary habit, alcohol consumption, and life style [33,34]. However, most of these information have not been collected. Thus, the confounding effect of these factors could not be thoroughly evaluated. Second, the subgroup analysis was stratified by first dialysis age and follow-up time, while these could not be analyzed in the general population.

## 5 Conclusions

In summary, our data supported the need of monitoring the development of cancer among ESRD patients undergoing dialysis, especially among younger patients and patients undergoing shorter time of dialysis. However, the current evidence was retrieved from retrospective studies, and it is recommended to conduct high-quality studies with larger sample size for verification.

## Appendix

**Table A1:** Selection strategy and results in PubMed (the retrieval time: 2021/02/19)

Search	Query	Items found
#1	“dialysis”[MeSH Terms] OR “dialysis”[tiab] OR “renal dialysis”[MeSH Terms] OR “renal dialysis”[tiab] OR “hemodialysis”[tiab]	195,998
#2	“neoplasms”[MeSH Terms] OR “neoplasms”[tiab] OR “cancer”[tiab] OR “cancers”[tiab] OR “carcinoma”[MeSH Terms] OR “carcinoma”[tiab] OR “carcinomas”[tiab]	4,038,934
#3	#1 AND #2	8,098
#4	“cross sectional studies”[MeSH Terms] OR “cross sectional studies”[tiab] OR “cohort studies”[MeSH Terms] OR “cohort study”[tiab] OR “longitudinal studies”[MeSH Terms] OR “longitudinal study”[tiab] OR “prospective”[tiab] OR “prospectively”[tiab] OR “retrospective studies”[MeSH Terms] OR “retrospective study”[tiab] OR “case-controlled”[tiab] OR “case control studies”[MeSH Terms] OR “case control study”[tiab] OR “risk”[tiab] OR “incidence”[tiab]	4,817,105
#5	#3 AND #4	2,747

**Table A2:** Selection strategy and results in Embase (the retrieval time: 2021/02/19)

Search	Query	Items found
#1	(‘dialysis’/exp OR dialysis:ti,ab OR ‘renal dialysis’/exp OR ‘renal dialysis’:ti,ab OR ‘hemodialysis’/exp OR ‘hemodialysis’:ti,ab)	228,807
#2	(‘neoplasms’/exp OR ‘neoplasms’:ti,ab OR ‘cancer’/exp OR ‘cancer’:ti,ab OR ‘cancers’:ti,ab OR ‘carcinoma’/exp OR ‘carcinoma’:ti,ab OR ‘carcinomas’:ti,ab)	4,542,487
#3	#1 AND #2	17,060
#4	(‘cross-sectional study’/exp OR ‘cross-sectional study’:ti,ab OR cohort:ti,ab OR prospective:ti,ab OR retrospective:ti,ab OR ‘case-controlled’:ti,ab OR risk:ti,ab OR ‘incidence’/exp OR incidence:ti,ab)	5,022,690
#5	#3 AND #4	6,506

**Table A3:** Selection strategy and results in the Cochrane library (the retrieval time: 2021/02/19)

Search	Query	Items found
#1	MeSH descriptor: [Dialysis] explode all trees	24
#2	MeSH descriptor: [Renal Dialysis] explode all trees	5,178
#3	(“dialysis” OR “renal dialysis” OR “hemodialysis”):ti,ab,kw (Word variations have been searched)	18,825
#4	#1 OR #2 OR #3	19,033
#5	MeSH descriptor: [Neoplasms] explode all trees	80,788
#6	MeSH descriptor: [Carcinoma] explode all trees	13,713
#7	(“neoplasm” OR “neoplasms” OR “cancer” OR “cancers” OR “carcinoma” OR “carcinomas”):ti,ab,kw (Word variations have been searched)	193,852
#8	#4 AND #7	490
#9	#8 in Trials	477
